# Fecal Calprotectin Elevations Associated with Food Intolerance/Malabsorption Are Significantly Reduced with Targeted Diets

**DOI:** 10.3390/nu15051179

**Published:** 2023-02-27

**Authors:** Wolfgang J. Schnedl, Simon Michaelis, Dietmar Enko, Harald Mangge

**Affiliations:** 1 Department of Internal Medicine, Auenbruggerplatz 15, A-8036 Graz, Austria; 2 General Internal Medicine Practice, Dr. Theodor Körnerstrasse 19b, A-8600 Bruck, Austria; 3 Institute of Clinical Chemistry and Laboratory Medicine, Hospital Hochsteiermark, Vordernberger Straße 42, A-8700 Leoben, Austria; 4 Clinical Institute of Medical and Chemical Laboratory Diagnosis, Medical University of Graz, Auenbruggerplatz 30, A-8036 Graz, Austria

**Keywords:** fecal calprotectin, irritable bowel syndrome, inflammatory bowel disease, fructose malabsorption, histamine intolerance, lactose intolerance

## Abstract

Inflammatory bowel disease (IBD) involves two clinically defined entities, namely Crohn’s disease and ulcerative colitis. Fecal calprotectin (FCAL) is used as a marker to distinguish between organic IBD and functional bowel disease in disorders of the irritable bowel syndrome (IBS) spectrum. Food components may affect digestion and cause functional abdominal disorders of the IBS spectrum. In this retrospective study, we report on FCAL testing to search for IBD in 228 patients with disorders of the IBS spectrum caused by food intolerances/malabsorption. Included were patients with fructose malabsorption (FM), histamine intolerance (HIT), lactose intolerance (LIT), and *H. pylori* infection. We found elevated FCAL values in 39 (17.1%) of 228 IBS patients with food intolerance/malabsorption and *H. pylori* infection. Within these, fourteen patients were lactose intolerant, three showed fructose malabsorption, and six had histamine intolerance. The others had combinations of the above conditions: five patients had LIT and HIT, two patients had LIT and FM, and four had LIT and *H. pylori*. In addition, there were individual patients with other double or triple combinations. In addition to LIT, IBD was suspected in two patients due to continuously elevated FCAL, and then found via histologic evaluation of biopsies taken during colonoscopy. One patient with elevated FCAL had sprue-like enteropathy caused by the angiotensin receptor-1 antagonist candesartan. When screening for study subjects concluded, 16 (41%) of 39 patients with initially elevated FCAL agreed to voluntarily control FCAL measurements, although symptom-free and -reduced, following the diagnosis of intolerance/malabsorption and/or *H. pylori* infection. After the initiation of a diet individualized to the symptomatology and eradication therapy (when *H. pylori* was detected), FCAL values were significantly lowered or reduced to be within the normal range.

## 1. Introduction

Crohn’s disease and ulcerative colitis are inflammatory bowel diseases (IBD), which are idiopathic disorders causing inflammation in the gastrointestinal (GI) tract. GI endoscopy, including histologic evaluation of the GI mucosa, are the golden standard, but is an invasive test for the diagnosis, management, and surveillance of IBD. Additionally, endoscopy is associated with considerable costs, perils, and burdens to patients and health systems [1]. Therefore, precise non-invasive tests, such as biomarkers and radiological examinations, are being sought [2]. Fecal calprotectin (FCAL) determinations are hence clinically recognized to help in the diagnosis, management and treatment monitoring of IBD [3]. FCAL is produced by inflammatory cells, mainly neutrophils entering the intestinal lumen from the GI mucosa during intestinal inflammation. Its levels significantly correlate with clinical or endoscopic disease activity. Additionally, FCAL has evolved as a widely used marker to distinguish between organic inflammatory (IBD) and functional bowel disease in disorders of the irritable bowel syndrome (IBS) spectrum [4]. Symptoms of the disorders of the IBS spectrum include various, usually postprandial, abdominal complaints. The exact pathophysiology of IBS disorders across the spectrum remains unclear.

Epidemiological and clinical studies indicate that specific nutrients in westernized diets pose a risk for the development and unfavorable disease courses of IBD [2]. So far, therapeutic dietary efforts in IBD and in functional GI disease (as disorders of the IBS spectrum) have not been successful [5]. However, food intolerance/malabsorption needs to be considered in patients with disorders of the IBS spectrum. Sugars (fructose, lactose), proteins (gluten), biogenic amines (histamine) [6] and infection with *Helicobacter pylori (H. pylori)* may cause symptoms across the IBS spectrum [7]. Accordingly, unabsorbed and undigested foods enter the colon, where the microbiota ferment them as bacterial substrate, which in turn may cause functional, non-specific, non-allergic GI symptoms [8] across the IBS spectrum.

Although the clinical usefulness of FCAL determinations has been demonstrated, there are limitations [9]. Our results show that elevated FCAL levels may be associated with food intolerance/malabsorption and combinations thereof, with or without *H. pylori* infection, and that FCAL may be lowered significantly with individualized diets adapted to the symptomatology.

## 2. Methods

At presentation, all patients had symptoms of IBS spectrum disorders; therefore, tests for food intolerances/malabsorption and *H. pylori* were performed. The patients’ complaints included diarrhea, soft stools, bloating, and chronic abdominal pain. The initial stool tests for FCAL determinations were performed before patients received their individual diagnoses and in advance of their change in dietary behavior. In patients with initially elevated FCAL, control FCAL measurements were arranged via invitation by phone 3 to 6 months after diagnosis and following dietary behavior change.

In the morning at the first presentation, blood drawings were performed after overnight fasting (>12 h) and hydrogen (H_2_) lactose breath tests were started. An H_2_ breath test was used at first presentation to examine whether the patient had LIT, and at second presentation to examine whether the patient had FM. Lactose H_2_ breath tests were performed with Gastrolyzer (Bedfont Scientific Inc., Kent, UK) using 200 mL water and, 50 g of lactose after fasting. End-expiratory exhalation of H_2_ was measured every 30 minutes (min) for a period of 120 min. Blood glucose was determined with a glucose oxidase method, every 60 min for a period of 120 min. A lactose intolerance was diagnosed when the H_2_ increased > 20 parts per million (ppm) or the blood glucose increase was <20 mg/dL. FM was tested using H_2_ breath tests and with a drink containing 25 g of fructose (Gastrolyzer, Bedfont Scientific Inc., Kent, UK). FM was present with the exhalation of H_2_ > 20 parts per million (ppm) compared to fasting H_2_. Before, the breath tests no antibiotics or laxatives were allowed and no colonoscopy was performed within 4 weeks prior to the breath test. 

The Liaison XS chemiluminescence technology immunoassay was used to determine the quantitative measurement of calprotectin in stool samples (DiaSorin, Centralino, Italy). To identify HIT, systematic anamneses with a standardized questionnaire concerning abdominal complaints of HIT, and the response to a histamine-reduced diet was used. Diamine oxidase (DAO) in serum was determined using the radio extraction assay DAO Rea 100 (Sciotec Diagnostic Technologies, Tulln, Austria). Antibodies against tissue transglutaminase were determined using anti-tTG IgA ELISA to consider celiac disease (Euro Diagnostica AB, Malmö, Sweden). An enzyme-linked IgA immunosorbent assay, anti-tTG IgA ELISA (Serion, Würzburg, Germany), or histologic evaluation of gastric mucosal biopsies were used to detect *H. pylori.* The statistical analyses were performed using SPSS 26.0 statistical software (SPSS Inc., Chicago, IL, USA).

## 3. Results

In a retrospective analysis of outpatients’ charts, we identified 228 FCAL determinations in consecutive patients (mean age 46 years, range 18–82 years; 72 male, 156 female) with food intolerance/malabsorption and/or *H. pylori*. After the diagnosis of individual food intolerance/malabsorption, patients received written information regarding their personal food malabsorption/intolerance or the combinations thereof. A registered dietician helped improve a diet individually tailored to each patient’s symptomatology.

**Figure 1:**
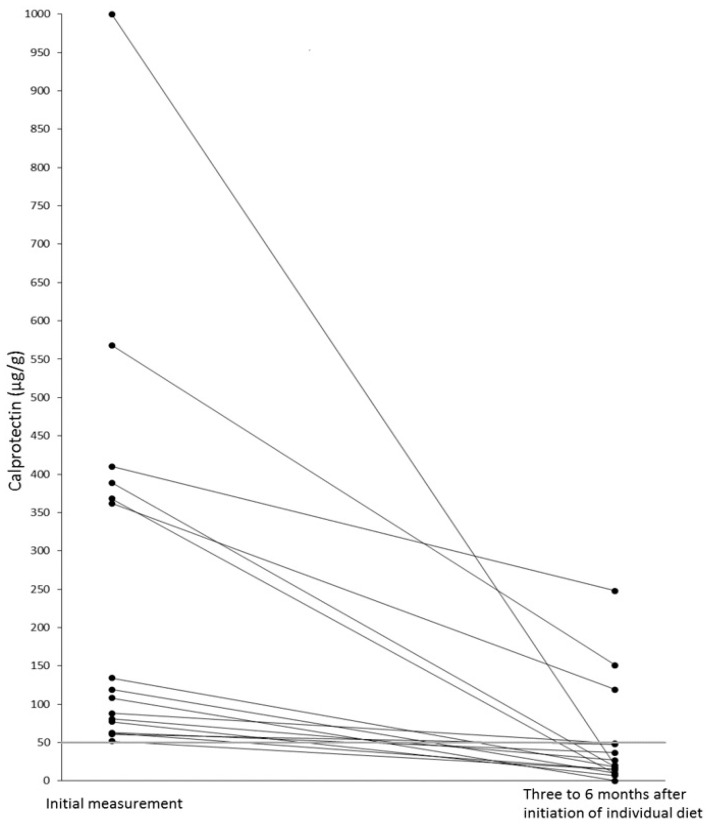
Reduction of elevated FCAL in 14 symptom-free patients with food intolerance/malabsorption after initiation of individualized diets. Improved symptoms in two patients with an initial diagnosis of LIT and a lactose-free diet. In these, a subsequent colonoscopy histologically diagnosed IBD.

Table 1 presents the diagnosed food intolerance/malabsorption, their various combinations and/or *H. pylori* infections with the number and percentage of patients with elevated FCAL. After contact via phone, 16 patients with previously elevated FCAL > 50 μg/g, came for a control of FCAL after 3 to 6 months. Figure 1 shows the significant reduction in FCAL levels after initiation of an individualized diet in these patients diagnosed with food intolerance/malabsorption. White blood cell counts, including neutrophil granulocytes, were within the normal range in all patients.

As a nonparametric statistical test for pairwise comparison of FCAL evaluations, the Wilcoxon test demonstrated a significant statistical difference (*p* < 0.001) from the initial measurement to the second control FCAL test.

Fourteen patients with initially elevated FCAL were symptom-free following their individualized diets and did not want to control their FCAL, four declined, and three were not contacted. One patient had sprue-like enteropathy due to candesartan, and other causes for IBS symptoms, including colonoscopy with biopsies, food intolerance/malabsorption, and *H. pylori* were ruled out. FCAL concentration was measured 105 μg/g and the cessation of candesartan therapy led to a full recovery [10], which caused him to disagree with further FCAL control. IBS symptoms improved in two patients with an initial diagnosis of LIT, but some complaints persisted, despite good adherence to a lactose-free diet. When the control FCAL value was >120 μg/g, a subsequent colonoscopy histologically diagnosed IBD, and medical treatment was initiated. In one asymptomatic patient, FCAL levels were still elevated but significantly lower, and colon biopsies revealed no abnormalities. This was explained by partial noncompliance with dietary recommendations. Patients aged >50 years were screened via colonoscopy and no pathologies were present. Nonetheless, a selection bias could not be excluded from our single-center experience. Given the low number of patients in the current study, larger patient studies are required in order to determine the clinical significance of our findings.

The numbers and percentages of individual food intolerance/malabsorption and *H. pylori* infections in 39 patients with elevated FCAL levels showed that 26 patients had LIT (66.7%), 13 had HIT (33.3%), 8 had FM (20.5%), and 6 patients had an *H. pylori* infection (15.4%). One patient had a sprue-like enteropathy.

## 4. Discussion

Originally, IBS was determined according to the Rome consensus meetings, but performed validation studies advocate a simpler determination of classic IBS symptoms [11]. Nonetheless, these complaints, usually not alarming in mainly young patients and women, now include IBS diarrhea, IBS constipation, functional diarrhea, functional constipation, chronic functional abdominal pain, and bloating [5]. Parallels in the pathophysiology of organic IBD and IBS are being sought, even though scientific evidence for such a link has been scarce. Particularly in patients with seemingly low-grade inflammation, there seems to be considerable overlap between organic IBD and IBS [12]. Clinical guidelines recommend FCAL evaluation in patients with suspected IBS or functional GI complaints to exclude the presence of IBD [13,14].

FCAL concentrations reflect the activity of inflammatory cells, including neutrophils, in the intestinal mucosa. It is clinically used as a marker to quantify intestinal inflammation, as its concentrations significantly increase in patients with IBD. Subsequently, the correlation of FCAL with clinical disease activity helps to distinguish suspected or confirmed IBD [4]. This was evident in two patients described here, because besides LIT, an IBD was found via histologic evaluation of biopsies taken during colonoscopy. Their FCAL levels were already significantly reduced due to good adherence to a lactose-free diet before IBD was diagnosed.

Nonetheless, the gut infiltration of neutrophil leucocytes in IBD is not specific. Overall, the exact etiology of IBD as of IBS is still uncertain. It is suggested that abnormal activation of the immune system, genetic susceptibility, and altered intestinal flora—due to mucosal barrier defects—may play a role in the development of these disorders. Nevertheless, both conditions influence the gut microbiota [15]. Generally, the dietary influence on the course of human IBD and IBS remains to be determined. Seemingly, some dietary supplements, such as vitamin D, fatty acids, and zinc, disrupt the intestinal bacterial flora, possibly affecting FCAL [16]. In addition, FCAL concentrations may vary throughout the day and daily [9].

In particular, lactose intolerance (LIT) appears as a documented reason for IBS spectrum complaints [8]. Relationships between intestinal lactase, the lactose-degrading enzyme, with small intestinal microbiota were recently reported [17]. In histamine intolerance (HIT), an influence on digestion with intestinal dysbiosis was shown [18]. Increasing the amount of ingested fructose causes several health issues, and has been shown to impact gut microbiota [19]. The influence of *H. pylori* (which is classified as a carcinogen) on GI microbial diversity and composition [20] and on exhaled hydrogen in lactose breath tests has been described [21]. However, we did not find elevated FCAL in *H. pylori*-only patients. Patients infected with *H. pylori* had high FCAL levels only in combination with food intolerance/malabsorption.

To date, dietary management in disorders of the IBS spectrum is insufficient. Approximately half of adult LIT patients not only require reduced lactose intake, but also need additional diet adjustments to improve their GI symptoms [22]. However, this was shown to be related to the presence of additional food intolerance/malabsorption [6]. Most patients are unable to attribute their IBS spectrum symptoms to a symptom-causing food or food ingredient. Hence, single food intolerances and various combinations of food intolerance/malabsorption, including evaluation for infection with *H. pylori*, need to be considered for etiological clarification [23]. In this study, we demonstrated that elevated FCAL levels were associated with food intolerance/malabsorption, and combinations thereof, with or without *H. pylori* infection, and that the introduction of individualized diets significantly lowered FCAL values.

FCAL is clinically used to guide diagnostic and therapeutic decisions as a well-studied inflammatory biomarker due to its stability, assay reproducibility, and low cost. Currently, FCAL concentrations < 50 μg/g are considered normal. FCAL concentrations higher than 200 μg/g may indicate the presence of an endoscopic active state of IBD [24]. However, we demonstrated that FCALs up to 1.000 μg/g could return to <50 μg/g with good dietary compliance, resulting in symptom-free food intolerance/malabsorption patients.

There is a complex relationship between diets, symptoms, and intestinal inflammation [25], and the pathogenesis of IBS seems not limited to the colon, but may involve the entire intestinal tract. This is following the fact that IBS symptoms are related to food intake in a large proportion of patients [26]. A certain relationship has been demonstrated between intestinal lactase and the microbiome of the small intestine [16]. In children, small intestinal biopsies demonstrated the association of lactase with mucosal DAO activities [27]. Serum DAO values were shown to influence adults’ lactose tolerance breath test results [28]. It seems that the small intestine is involved in HIT and LIT food intolerances. Intestinal sugar absorption modulates the postprandial glycemic response involving GLUT-5 expression, which is reduced in FM [29]. However, further work, which should consider the intestinal tract as a whole, is required to answer questions regarding FCAL elevations caused by food intolerance/malabsorption.

## 5. Conclusions

We demonstrated that food intolerance/malabsorption might have caused mucosal infiltration of neutrophils in patients with elevated FCAL values. This inflammation seems to have caused abdominal symptoms of the IBS spectrum in food intolerance/malabsorption. Subsequently, in patients who were symptom-free due to good adherence to their individualized food-reduction and/or elimination diets (according to their individual diagnoses), FCAL levels were clearly reduced, or returned to within normal range. Targeted dietary intervention using reduction or exclusion diets for single or possibly combined food intolerance/malabsorption reduced intestinal inflammation and contributed to symptom relief with significant FCAL reduction.

## Figures and Tables

**Table 1:** FCAL determined in 228 patients with food intolerance/malabsorption and/or *H. pylori*.

Food Intolerance/Malabsorption	Number (*n*) of Patients	Percent (%) of Patients	Number (*n*) of Patients with High FCAL	Percent (%) of Patients with High FCAL
Number of patients	228	100	39	17.1
LIT-only	49	21.5	14	28.6
FM-only	22	9.6	3	13.6
HIT-only	29	12.7	6	20.7
*H. pylori*-only	10	4.4	0	0
LIT + FM	18	7.9	2	11.1
LIT + FM + HIT	21	9.2	1	4.8
LIT + HIT	34	14.9	5	14.7
LIT + *H. pylori*	14	6.1	4	28.6
LIT + HIT + *H. pylori*	4	1.7	0	0
LIT + FM + *H. pylori*	5	2.2	0	0
LIT + FM + HIT + *H. pylori*	2	0.9	0	0
FM + HIT	11	4.8	1	9.1
FM + HIT + *H. pylori*	5	2.2	0	0
FM + *H. pylori*	1	0.4	1	100
HIT *+ H. pylori*	2	0.9	1	50
Candesartan	1	0.4	1	100

LIT, lactose intolerance; FCAL, calprotectin; FM, fructose malabsorption; HIT, histamine intolerance; *H. pylori*, *Helicobacter pylori*.

## Data Availability

The data that support the findings of this study are available from the corresponding author upon reasonable request.

## Abbreviations

Celiac disease, CD; diamine oxidase, DAO; fecal calprotectin, FCAL; fructose malabsorption, FM; gastrointestinal, GI; *Helicobacter pylori*, *H. pylori*; histamine intolerance, HIT; irritable bowel syndrome, IBS; inflammatory bowel disease, IBD; lactose intolerance, LIT.
